# Utility of the Biosynthetic Folate Pathway for Targets in Antimicrobial Discovery

**DOI:** 10.3390/antibiotics3010001

**Published:** 2014-01-21

**Authors:** Christina R. Bourne

**Affiliations:** Department of Veterinary Pathobiology, Oklahoma State University, 250 McElroy Hall, Stillwater, OK 74078, USA; E-Mail: cbourne@ou.edu; Tel.: +1-405-325-5348; Fax: +1-405-325-6111

**Keywords:** folate pathway, antimicrobial, polypharmacology

## Abstract

The need for new antimicrobials is great in face of a growing pool of resistant pathogenic organisms. This review will address the potential for antimicrobial therapy based on polypharmacological activities within the currently utilized bacterial biosynthetic folate pathway. The folate metabolic pathway leads to synthesis of required precursors for cellular function and contains a critical node, dihydrofolate reductase (DHFR), which is shared between prokaryotes and eukaryotes. The DHFR enzyme is currently targeted by methotrexate in anti-cancer therapies, by trimethoprim for antibacterial uses, and by pyrimethamine for anti-protozoal applications. An additional anti-folate target is dihyropteroate synthase (DHPS), which is unique to prokaryotes as they cannot acquire folate through dietary means. It has been demonstrated as a primary target for the longest standing antibiotic class, the sulfonamides, which act synergistically with DHFR inhibitors. Investigations have revealed most DHPS enzymes possess the ability to utilize sulfa drugs metabolically, producing alternate products that presumably inhibit downstream enzymes requiring the produced dihydropteroate. Recent work has established an off-target effect of sulfonamide antibiotics on a eukaryotic enzyme, sepiapterin reductase, causing alterations in neurotransmitter synthesis. Given that inhibitors of both DHFR and DHPS are designed to mimic their cognate substrate, which contain shared substructures, it is reasonable to expect such “off-target” effects. These inhibitors are also likely to interact with the enzymatic neighbors in the folate pathway that bind products of the DHFR or DHPS enzymes and/or substrates of similar substructure. Computational studies designed to assess polypharmacology reiterate these conclusions. This leads to hypotheses exploring the vast utility of multiple members of the folate pathway for modulating cellular metabolism, and includes an appealing capacity for prokaryotic-specific polypharmacology for antimicrobial applications.

## 1. Introduction

The application of antibiotic treatments has revolutionized health care, but the antimicrobial development pipeline is quickly becoming depleted and new therapeutics are badly needed [1,2,3,4]. During this timeframe, healthcare has also experienced an explosive growth in the number of strains of drug-resistant bacteria, exacerbating the need for new antimicrobials [5,6]. Antibiotic drug development was stalled for almost two decades, in large part due to unforeseen problems with newer and innovative approaches [7]. The advent of large-scale genomics prompted target-based efforts, where specific proteins essential to bacterial growth were employed in high-throughput screening efforts. While this did produce “hits”, the translation to whole cells screens resulted in a lack of permeability or solubility in almost every case [3,4,8].

It is increasingly recognized that the paradigm of a single drug inhibiting a single target is oversimplified, and in reality most compounds exhibit a spectrum of pharmacology [9,10,11,12]. Efforts for identifying targets have led investigators to explore metabolic pathways, and computational efforts have delineated a predominance of a select few chemotype scaffolds that also display previously unappreciated polypharmacology [10,13,14,15,16]. The recognition of multiple mechanisms of action (MOAs) is being taken advantage of by re-purposing of existing therapeutics for so-called “off label” or “secondary” effects [17,18]. To date, however, efforts to re-purpose U.S. Federal Drug Administration approved drugs to leverage discovered off-target effects have not progressed quickly enough to meet current challenges. The application of sub-minimum inhibitory concentrations (MIC) levels of antibiotic is known to affect cellular targets not necessarily related to the therapeutic MOA, consistent with a polypharmacologic approach [19,20,21]. With the computational studies of metabolic pathways has come the recognition that many members of a pathway bind to structurally similar endogenous substrates and products [22,23,24,25]. When an inhibitor to one of these members is designed to mimic the endogenous ligand, the probability of cross-reactions with other pathway members increases dramatically. By targeting multiple cellular systems the efficiency of bacterial resistance mechanisms would be greatly diminished [11,24,26].

A typical example of polypharmacology can be found in the folate pathway [22,26]. While there has been much work within the human folate pathway, little is published regarding polypharmacology of the bacterial folate metabolism pathways. Current approaches employing anti-folates rely on inhibiting dihydrofolate reductase (DHFR), which displays sequence variations allowing selective inhibition, and dihydropteroate synthase (DHPS), an enzyme lacking in higher eukaryotes [27,28,29,30,31,32,33,34]. Extensive work on chemo-centric groupings has highlighted the overlap among folate pathway members with respect to ligand structures [35,36,37,38]. However, much of this work is focused on pathway members with known inhibitors, such as DHFR and DHPS, and, thus, other members in the folate pathway remain uncharacterized with regards to their potential for overlapping inhibition. Computational studies have found that ligands for one particular folate enzyme are likely to also bind to other folate compounds. In addition, inhibitors for a given enzyme in the folate pathway have a high probability of binding to other folate pathway members [22]. While specific examples of anti-folate inhibitors binding multiple proteins in the pathway exist (for example, Pemetrexed [39]), there remains a gap in systematic experimental evidence to support these activities. Specific links have been made between inhibitors of dihydrofolate reductase (DHFR) and other downstream enzymes in eukaryotes: phosphoribosyl-glycinamide formyl-transferase (GART), phosphoribosyl-aminoimidazole-carboxamide formyl-transferase (AICART), and thymidine synthase (TS) [35,39,40,41]. The folate biosynthetic pathway found upstream of DHFR is unique to prokaryotes and lower eukaryotes, which cannot acquire folate from their environment. There are efforts being made to target multiple binding sites within the same folate pathway enzyme (“bi-substrate” inhibitors) [42,43,44,45]; however, efforts to target multiple individual binding sites of enzymes upstream of prokaryotic DHFR are limited [46].

Numerous previous reviews of the folate biosynthesis pathway are available [27,34,47,48,49,50,51]. The current review will set out to characterize the antibacterial therapeutic potential of targets in folate metabolism upstream of DHFR with an emphasis on the overlap of binding potentials of individual targets. This is to address the hypothesis that, armed with the current state of knowledge, a minimalistic inhibitor could be designed to modulate multiple but specific targets within this pathway in pathogens. The main hurdle will be species selectivity, such that bacterial pathogens can be targeted while minimizing deleterious actions against the eukaryotic host. Homology between prokaryotes and eukaryotes is limited by focusing on the biosynthetic pathway upstream of DHFR, which is not present in organisms such as eukaryotes. A table listing the available entries for bacterial folate biosynthesis enzymes from the Protein Databank (as of October 27, 2013), as discussed further below, is included for reference purposes as Supplementary Table S1 [52]. These listings include the organism used as the source of the enzyme, the complexed ligands and the primary citations in efforts to facilitate further studies.

### Pathways for Folate Production and Utilization

The DHFR enzyme is a central player in the folate pathway that links divergent folate synthesis or acquisition mechanisms to the production of tetrahydrofolate (THF). The THF product serves as a critical co-factor by shuttling methyl and formyl groups utilized in one-carbon transfer reactions, which are required for synthesis of purines, amino acids, S-adenosylmethionine, and formyl-methionine [51,53]. As there is no redundancy in the reaction carried out by DHFR, it is an obligate and critical metabolic node. Inhibition of DHFR directly halts cellular replication by starving the cell of needed cellular precursors; its effectiveness has been proven in anti-cancer, anti-plasmodial, and anti-bacterial treatments [27,34,50].

Most of the one-carbon transfer reactions downstream of DHFR are redundant or highly conserved, but the upstream pathways are divergent between eukaryotes and prokaryotes. Included in the upstream biosynthesis are reactions that form the pterin component of folate. Pterin synthesis in eukaryotes utilizes, among others, the enzyme sepiapterin reductase to produce tetrahydrobiopterin [30]. This product is critical for proper signaling in nerves and has an important role in the homeostasis of brain chemistry [54]; mutations in this protein are associated with phenylketonuria and related metabolic syndromes [55,56]. Prokaryotes lack this pathway, but instead synthesize the pterin portion of folate *de novo* from guanosine triphosphate (GTP), *p*-aminobenzoic acid (pABA) and glutamate. This flux passes through a series of seven enzymes unique to bacteria, protozoa, and some lower eukaryotes [27,34,51]. The folate pathway is heavily pursued for drug targets due to its obligate nature and to the very selective profile of the enzymes. Of note is the reliance on the shikimate pathway for production of GTP, which contains the target of the herbicide glyphosate [57,58,59,60,61]. In some organisms, including yeast and plants, there also exists an overlapping pathway that can salvage folate, such as malarial parasite from the serum of the infected host [48,62].

## 2. Results and Discussion

### 2.1. Enzymes for the Biosynthesis of Folate: 1. Guanine Cyclohydrolase (GCYH I)

**Figure 1:**
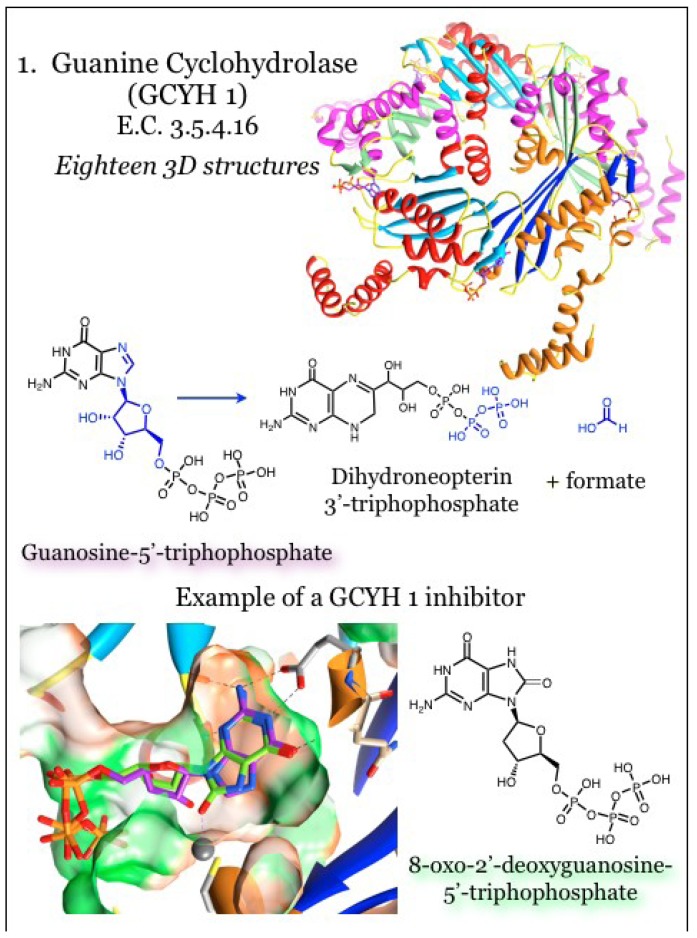
Guanine cyclohydrolase (GCYH I) catalyzes a reaction utilizing GTP and producing dihydroneopterin 3'-triphosphate. Coordinates for GCYH I were taken from PDB ID 1WUR [56] and 4DU6 [69] with GTP (purple). Pentameric subunits can be distinguished by altered colors for beta-strands (blue, dodger blue, cyan) and alpha-helices (orange, red, magenta). A catalytic zinc atom is represented by a larger grey sphere, and is coordinated with a conserved Cys residue. The guanine base resides in a relatively hydrophobic pocket (orange surface) with polarity limited to the nitrogen atoms of the heterocycle. Conserved interactions, including between the guanine base and an acidic residue, are indicated.

In bacteria, that pathway to pterin synthesis is initiated with GTP, which undergoes ring opening, rearranging and reclosing to form dihydroneopterin 3'-triphosphate via guanine cyclohydrolase I (GCYH I, E.C. 3.5.4.16, Figure 1), encoded by the *folE* gene [51,56,63,64,65,66]. The protein is composed of a pentameric beta-barrel surrounded by alpha helices, and the active site requires contributions from three subunits [56,63,64,65,66]. The enzyme requires a zinc cation, which is coordinated by two cysteine residues, one histidine residue, and ordered water molecules [56,63]. Derivatives of 8-oxoguanine competitively inhibit the bacterial enzyme *in vitro* by mimicking the transition state configuration [56]. The guanine moiety of 8-oxo-GTP was found inserted into a cavity of the protein utilizing interactions similarly to pterin-binding proteins. Polar groups within guanine ring are hydrogen bonded within the binding site, and in particular interact in a characteristic fashion with an acidic residue [56,63]. The triphosphate group was coordinated at the periphery of the binding site, which is basic in nature to complement the charged phosphate moieties [56].

Another enzyme possessing GCYH I activity has been described, and its gene has been named *folE2* [67]. This enzyme has no shared sequence with GCYH I described above, is prokaryotic specific, and is present in most *Archaea* and in bacteria that frequently lack the canonical GCYH I [68]. This highlights a potential Achilles heel for exploitation in antibacterial-specific targets. The overall T-type protein fold is maintained in a tetrameric arrangement, with two eight-stranded antiparallel beta-sheets pairing to form a 16-stranded beta barrel that is surrounded on each side with alpha helices. The tetramer further oligomerizes by forming a homodimer, yielding an octameric arrangement with D4 symmetry. The extrapolated binding cavity for the guanine moiety within this GCYH Ib enzyme is more shallow than in GCYH I, but the acidic Glu residue that coordinates the nitrogen groups is present. The basic patch to neutralize the phosphate groups is maintained, although the residues themselves are contributed by different subunits between GCYH I and GCYH Ib. While there are differences noted in the specific interactions, the overall pose of the guanine is the same between the two GCYH enzymes [68].

### 2.2. Enzymes for the Biosynthesis of Folate: 2. Dihydroneopterin Hydrolase (DHNTPase)

**Figure 2:**
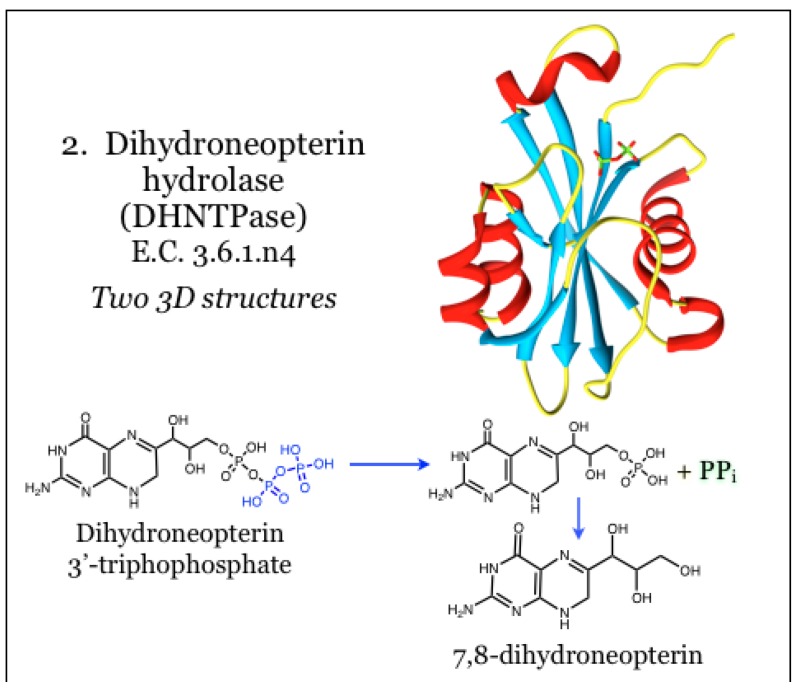
Dihydroneopterin hydrolase possesses pyrophosphatase activity for dihydroneopterin 3'-triphosphate, and in bacteria is carried out by a Nudix NTP hydrolase. Coordinates for DHNTPase were taken from PDB ID 2O1C, as complexed with pyrophosphate (green), which yields feedback inhibition [70]. No structural models with substrate or information on inhibitors are available.

The product of GCYH, 7,8-dihydroneopterin triphosphate, is then acted upon by a poorly characterized pyrophosphatase, called dihydroneopterin hydrolase (DHNTPase, E.C. 3.6.1.n4), to yield 7,8-dihydroneopterin. The identity of this required enzymatic activity has been questioned, and as such it has generally been excluded from therapeutic targeting efforts [27,34,51]. The gene for this activity has been identified as *nudB* in bacteria [48,70] and *folQ* in *Lactobacillus* and plants [48,70]. The product of the *nudB* gene, Nudix NTP hydrolase, catalyzes the removal of pyrophosphate from dihydroneopterin triphosphate (Figure 2) [70]. The site of catalysis is housed on a mixed beta-sheet surrounded by alpha helices, and the catalytic mechanism requires coordination with divalent metal ions, such as magnesium. Some evidence indicates that loops overhanging the catalytic site are mobile and control access, likely in concert with catalytic events. A structural model of dihydroneopterin within the active site was generated and reportedly predicts an aromatic stack of the pterin heterocycle between two Phe residues. In addition, hydrogen bonding potential of the pterin was satisfied by interaction with mainchain atoms, as well as Ser and Glu residues. To date this is the only structural information available for *nudB* gene product [70].

### 2.3. Enzymes for the Biosynthesis of Folate: 3. Dihydroneopterin Aldolase (DHNA)

**Figure 3:**
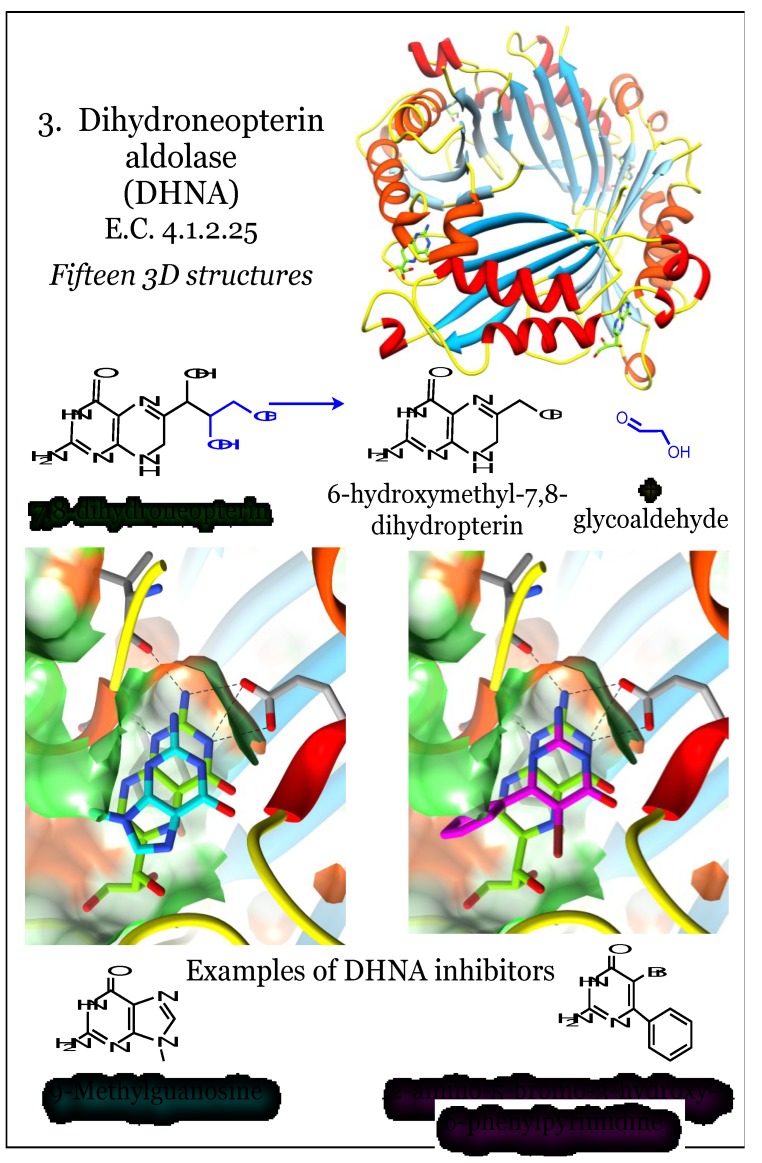
Dihydroneopterin aldolase (DHNA) utilizes 7,8-dihydroneopterin to produce 6-hydroxymethyl-7,8-dihydroneopterin and glycoaldehyde. Coordinates for DHNA were taken from PDB ID 2NM2, as complexed with a substrate neopterin compound (green) [71]. Additional structures were taken from PDB ID 1RRW, complexed with 9-methylguanosine (cyan), and 1RSI, complexed with a brominated pyrimidine (magenta) [77]. The four subunits comprising the tetrametric protein assembly can be distinguished by altered colors for beta-strands (dodger blue, sky blue) and alpha-helices (red, orange). The pterin moiety is bound in a relatively polar (green surface) cleft between two subunits. Conserved interactions, including between the aminopterin moiety and an acidic residue, are indicated.

When folate synthesis reactions are evaluated for conservation among bacterial species, the first pathway member that readily qualifies is the enzyme encoded by the *folB* gene, dihydroneopterin aldolase (DHNA, E.C. 4.1.2.25, Figure 3) [27]. This enzyme uses 7,8-dihydroneopterin as a substrate and cleaves a carbon-carbon bond in a stereospecific manner to produce 6-hydroxymethyl-7,8-dihydropterin and glycoaldehyde [71,72,73]. This is a unique aldolase enzyme, requiring neither Schiff base formation within the enzyme (Class I aldolases) or a zinc ion (Class II aldolases) for catalysis [71,73]. Further, it is reported to exhibit reversible epimerase activity, converting 7,8-dihydromonapterin to its own substrate 7,8-dihydroneopterin [74]. Its structure is composed of a four-stranded antiparallel beta sheet with two long helices along one side of each monomer (Figure 3). The protein oligomerizes into a tetramer, resulting in a 16-stranded beta barrel, with the longer alpha helices surrounding the outside. In some bacteria, such as *M. tuberculosis*, these tetramers dimerize to yield an allosterically regulated active octamer [72]. In other bacteria, such as *Streptococcus pneumonia*, the active site is fully formed even as a tetramer [74]. There has been some suggestion that the octamerization is artifactual and a result of high protein concentration [75]. There are known variations in the organization of DHNA; for example, this activity is encoded by two different domains (*fasA*, *fasB*) in *Pneumocystis carinii*, and these are fused to the 2-amino4-hydroxy-6-hydroxymethyldihydropteridine pyrophosphokinase (HPPK) and DHPS enzymes [76]. There is a report of a FolX protein from *E. coli* that is a paralog of DHNA [48].

The DHNA active site is composed of residues from two different monomers and located external to the beta barrel [71,72,73,74,77,78]. The binding of the pterin substrate is driven by at least three specific hydrogen bonds contributed by main chain polar atoms of the protein and by acidic Glu and/or Asp residues. These anchor the pyrimidine ring, and in addition the pocket can be heavily hydrated [73,74,77]. The hydrophobic faces of the heterocycle are sandwiched between hydrophobic residues from each monomer, including Val, Leu, Pro, and a conserved and critical Tyr residue. The catalytic portion of the site is distal from the pterin-docking cavity, and catalysis utilizes a highly conserved acidic residue (usually a Glu) and a Lys residue [74,77]. The DHNA enzyme has been pursued as an antimicrobial target, as well as potential herbicide target in plants [78]. Of identified inhibitors, they are exclusively based on pterin or pyrimidine analogs that can fulfill a defined spatial pattern of hydrogen bonds within the binding site [77]. In particular, a medicinal chemistry undertaking demonstrated the ability to extend the structure of the analogs beyond an anchoring pterin-like group, although their most potent *in vitro* inhibitor failed to elicit inhibition of bacterial growth [77]. At least one group has noted the conservation of interactions between DHNA with the pterin ligand and of DHFR and its pterin ligand [73].

The architecture of the DHNA active site is homologous to other pterin-binding proteins, and, in particular, with pyruvoyltetrahydropterin synthase (PTPS, E.C. 4.2.3.12), an apparently redundant enzyme that is present in some bacteria and in *Archaea* [49,79,80]. PTPS is also present in eukaryotes, although sequence homology is distinctly low [81]. Organisms that rely on PTPS for folate synthesis include many important mediators of pathogenesis, including *Leishmania* species [81], *Plasmodium falciparum* [79] and *Toxoplasma gondii* [81]. The organization of this enzyme is as a dimer of trimers to form an active hexamer. The pterin-binding site has contributions from three monomers, and the overall orientation is reminiscent of DHNA as the trimers compose a 12-stranded barrel that then undergo dimerization [82].

### 2.4. Enzymes for the Biosynthesis of Folate: 4. 2-Amino-4-hydroxy-6-hydroxymethyldihydropteridine pyrophosphokinase (HPPK)

**Figure 4:**
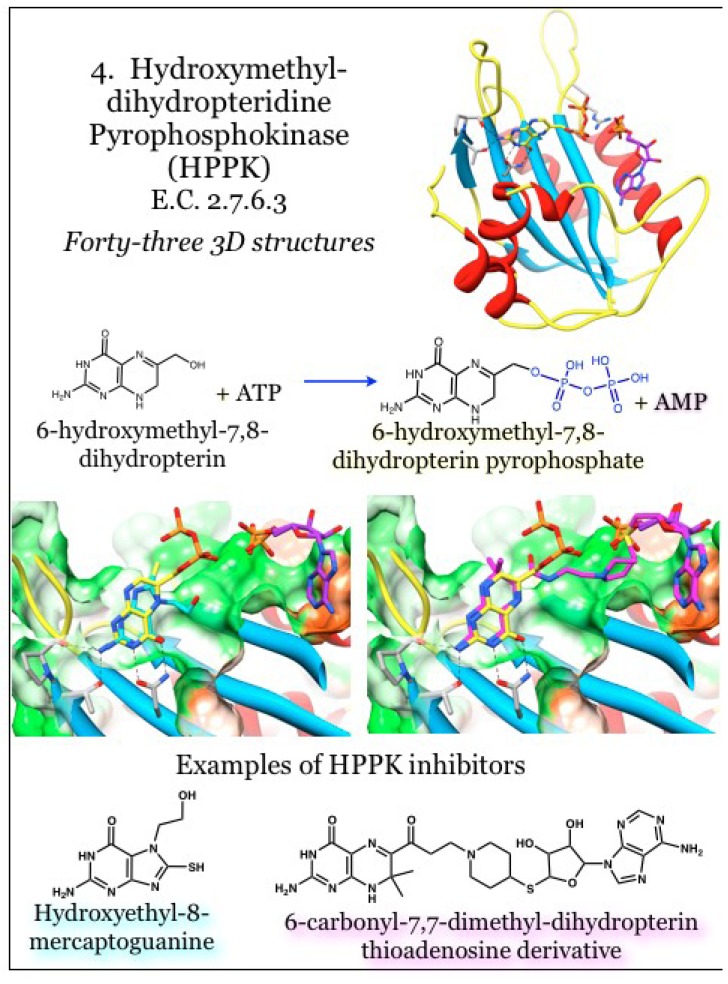
2-amino-4-hydroxy-6-hydroxymethyldihydropteridine pyrophosphokinase (HPPK) transfers a pyrophosphate unit from ATP onto 6-hydroxymethyl-7,8-dihydropterin. Coordinates for HPPK were taken from PDB ID 1RAO, complexed with the product 6-hydroxymethyl-7,8-dihydropterin pyrophosphate (yellow) and AMP (purple) [92]. The complexes with inhibitors were taken from PDB ID 4AD6 (hydroxyethyl-8-mercaptoguanine, cyan) and 3UDV (a bisubstrate linked dihydropterin thioadenosine, magenta) [84,95]. The pterin moiety is bound in a shallow and neutral (white surface) to polar (green surface) crevice. Conserved interactions with the aminopterin moiety are indicated.

The enzyme 2-amino-4-hydroxy-6-hydroxymethyldihydropteridine pyrophosphokinase, or HPPK (E.C. 2.7.6.3), catalyzes the transfer of a pyrophosphate group from an ATP donor onto 6-hydroxylmethyl-7,8-dihydropterin (HMDP), the product of the DHNA enzyme (Figure 4). This enzyme is among the most well studied pyrophosphate kinases, transferring a pyrophosphate unit at the beta phosphate rather than more typical kinase reaction utilizing the gamma phosphate. HPPK has been pursued as an antimicrobial target; however, identification of *in vitro* inhibitors has not translated into microbial growth inhibitors to date [43,44,51,83,84,85,86,87].

The monomeric protein contains a thioredoxin-like fold [86] composed of a four-stranded anti-parallel beta sheet with helices appended to each face. Connections between the elements of secondary structure provide three loops that undergo major structural transitions in response to binding and catalysis [88,89,90], although these changes are attenuated due to sequence differences between *E. coli* and other bacteria, such as *S. pneumoniae* [74] and *Y. pestis* [91]. The enzyme requires ordered binding, with a Mg^2+^-ATP unit binding first and triggering movement of a loop (L3) to more than 20 Å away from the binding site. This movement triggers ordering of critical Arg residues and also completes the formation of the substrate HMDP site [91,92]. The bound pterin moiety triggers a stabilization of the binding site and subsequent closure of loops L1 and L3 around the site; a Trp residue at the tip of L3 effectively seals the catalytic site from the bulk solvent [93]. The rate-limiting step is product release, which requires movement of L3 again away from the site. The entire catalytic cycle is believed to take six steps, including a transition state [94].

As with other pterin-binding enzymes from the biosynthetic folate pathway, the pterin moiety is bound within a cavity, with the catalytically active portion situated in a groove along the enzyme surface proximal to the ATP binding site [86,87]. The interactions between the protein and the pterin moiety include extensive hydrogen bonding and aromatic residues stacking on either face of the pterin heterocycle [86,94]. Investigations into inhibition revealed the most effective method was a to occupy both the pterin substrate and the adenosine co-factor sites, thus generating a “bisubstrate analog” [43,44,85,86]. Demonstrations have highlighted the requirement for the pterin moiety; a motif shared among the folate pathway members, such as in 8-mercaptoguanine [86]. The bridging phosphate groups could be replaced with other physiochemically desirable groups and the adenine base also had flexibility in docking orientation [92]. It is of note that 8-mercaptoguanine also shows competitive inhibition against DHPS, the next enzyme in the biosynthetic folate pathway [96]. Similarly, investigations with *Saccharomyces cerevisiae* HPPK-DHPS-fused enzyme revealed occupancy of both enzymes’ active sites by the same inhibitory pterin monophosphate analog compound [97]. These observations strongly support the supposition of inhibiting multiple enzymes from the folate biosynthetic pathway with a single therapeutic agent [86].

### 2.5. Enzymes for the Biosynthesis of Folate: 5. Dihydropteroate Synthase (DHPS)

**Figure 5:**
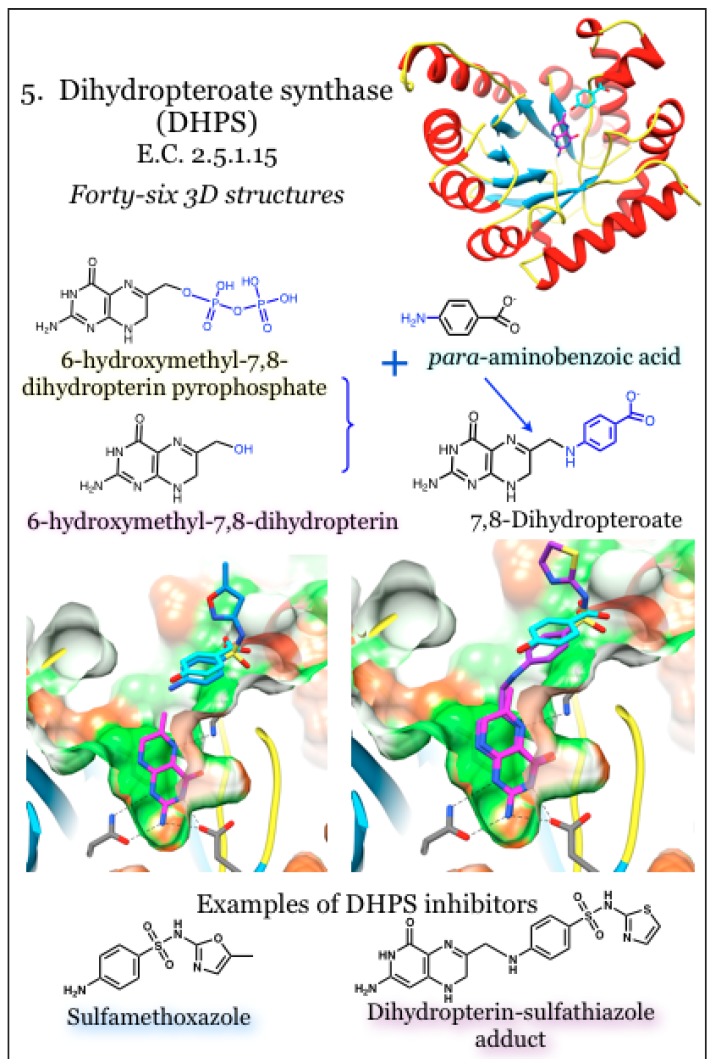
Dihydropteroate synthase (DHPS) joins 6-hydroxymethyl-7,8-dihydropterin pyrophosphate with *para-*aminobenzoic acid to produce 7,8-dihydropteroate. Coordinates for DHPS were taken from PDB ID 3TYB, complexed with the substrates 6-hydroxymehtyl-7,8-dihydropterin (magenta) and *para-*aminobenzoic acid acid (cyan) [101]. Structures with inhibitors are PDB ID 3TZF with sulfamethoxazole (blue) and 3TYE with a dihydropterin-sulfathiazole adduct (magenta) [101]. The pterin binding pocket is very polar (green surface); conserved interactions with the aminopterin moiety are indicated.

The enzyme dihydropteroate synthase (DHPS) is a dimeric triosephosphate isomerase-type (TIM)-barrel protein with eight parallel beta strands surrounded by eight alpha helices [42,98,99,100]. It catalyzes the condensation of 6-hydroxymethyl-7,8-dihydropterin pyrophosphate with *para*-aminobenzoic acid (pABA) to yield 7,8-dihydropteroate (Figure 5). DHPS is the bacterial target for one of the earliest introduced classes of synthetic antimicrobials, the sulfonamides [99,101]. Sulfonamide compounds are pABA analogs that in many, if not all, cases serve as an alternative substrate and yield a dead-end product [101,102,103,104,105]. Among the numerous off-target effects of sulfa drugs is the inhibition of mammalian sepiapterin reductase, critical to nerve cells for the production of tetrahydrobiopterin, which serves as a co-factor for neurotransmitter synthesis [54]. Mutations in the DHPS sequence are responsible for conferring resistance to sulfonamide therapeutics, and structurally these are located around the pABA binding site [42,96,100,106]. There is a required order of addition such that binding of the pterin substrates must precede pABA (or inhibitory analog) binding [99]. Early structures revealed high mobility for two loops known to be involved in binding to pABA and thus in conferring resistant to sulfa drugs. Pioneering studies using crystallized and catalytically active DHPS recently revealed a novel SN1-type mechanism that first bound the pterin pyrophosphate substrate, anchoring a mobile loop, which in turn formed the pABA site [101]. In the absence of the pterin substrate, the pterin-binding site is occupied by an Arg side chain. This Arg residue is displaced upon pterin binding and then re-locates to form part of the pABA site; as such, it is hypothesized to increase stability and provide for regulation of enzymatic activity [42]. The reaction is initiated by removing the pyrophosphate moiety independently from the condensation reaction, resulting in occupancy of unphosphorylated pterin substrates in the absence of pABA or an analog thereof [99,101]; magnesium is needed for stabilization and release of the pyrophosphate [101]. Free pyrophosphate can be used to enhance binding in the pABA pocket, such as the binding and inhibition by sulfonamide compounds [99].

In contrast to the sulfonamide-targeted pABA site, which is not preformed and requires ordering of mobile loops, the pterin site is buried and directed down within the core beta-barrel of the protein [98]. The sequence of the pterin-binding pocket is highly conserved among bacterial DHPS enzymes, as are the pterin-sites within enzymes involved in one-carbon transfers that use methyltetrahydrofolate as a co-factor [96,99,107]. Non-phosphorylated pterins, such as 6-(methylamino)-5-nitroisocytosine, are competitive inhibitors of DHPS *in vitro* [42]. Additionally, the basic pterin structure can provide weak inhibition that is improved by extension away from the pterin group and towards the surface of the beta barrel, which is into the pABA pocket [96,107]. Not surprisingly, a guanine-like compound was also found to be an inhibitor of DHPS by mimicking the pterin substrate [96]. Interactions with pterin groups, or analogs thereof, utilize hydrogen bonds formed with Asn, Lys and Asp residues, as well as polar interactions bridged by water molecules. Hydrophobic stacking interactions satisfy the heterocycle face with the guanidinium group of an Arg on one side and a cluster of small hydrophobic and aromatic residues on the other face [98,100,101,107]. A conserved Lys residue undergoes a conformational change to form hydrogen bonds with the pterin and, in so doing, comprises the base of the pABA site [96].

### 2.6. Enzymes for the Biosynthesis of Folate: 6. Dihydrofolate Synthetase (DHFS)

**Figure 6:**
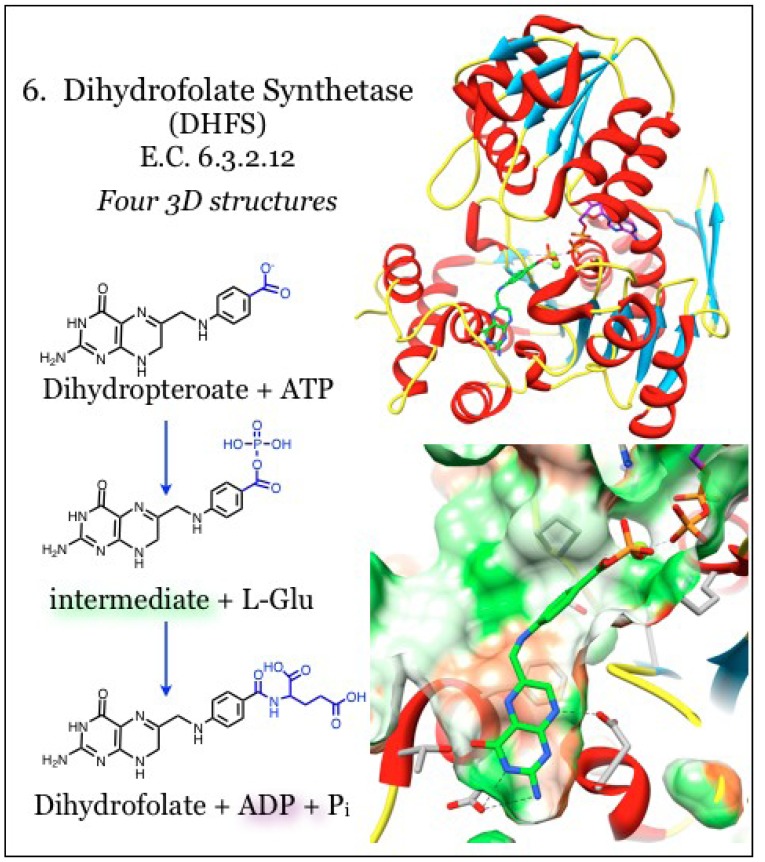
Dihydrofolate synthetase (DHFS) carries out glutamation of folate substrates. Coordinates for DHFS were taken from PDB ID 1W78, which is in complex with a phosphorylated intermediate of dihydropteroate (green) and ADP (purple) [108]. The polarity of the pocket coincides with the nitrogen atoms of the aminopterin (green surface), while the heterocycle face is paired with hydrophobic portions of the pocket (orange surface). Conserved interactions with the aminopterin moiety are indicated.

The substrate for dihydrofolate reductase (DHFR) enzyme is generated by the activity of dihydrofolate synthetase (DHFS, E.C. 6.3.2.12, Figure 6), which appends a single L-glutamate tail to the product of the DHPS enzyme, dihydropteroate, in an ATP-dependent reaction [51,108]. Further polyglutamylation can occur after reduction of the substrate by DHFR to tetrahydrofolate, and this polyglutamation activity is carried out by a more ubiquitous folylpolyglutamate synthetase activity (FPGS, E.C. 6.3.2.17) [109]. The appendage of negatively charged glutamate structures to the folate component prevents diffusion of the folate compound through cell membranes. This polar charge is also an important determinant for binding with enzymes downstream of DHFR, *i.e.*, the one-carbon transfer enzymes, making polyglutamation strategies particularly useful in anti-cancer therapeutics [53]. Despite the almost identical enzymatic activities of monoglutamation by DHFS and polyglutamation by FPGS, DHFS activity is found only in the folate biosynthesis pathway upstream of DHFR while the FPGS activity is only found downstream; however, in many bacteria both activities are encoded in the same *folC* gene [53,108,110,111,112].

The fold of either DHFS or FPGS conforms to the Mur synthetase superfamily and contains an ATPase domain and a Rossmann-fold domain, with the pterin binding site located between the two [113,114]. The ATP-binding site is conserved in a narrow channel between these domains and the binding is stabilized by interaction with water molecules [108]. The phosphate groups are coordinated with conserved residues and with two Mg^2+^ ions that neutralize the negative charges. In many structures, the ATP is already partially hydrolyzed even in the absence of the substrate. The pterin site is poorly ordered in the absence of substrate, but a mobile loop becomes ordered when the site is occupied [108]. The heterocycle of the pterin is secluded within a cavity and stacks between hydrophobic residues including Phe, Ala, Ile, and Leu. When the DHFS and FPGS activity is shared within a single protein, there is a separate inhibitory profile for each of the two enzymatic specificities [53,108,115]. The FPGS substrate contains additional functional groups on the pterin that require a larger binding pocket than what is found in DHFS enzymes. However, in bacterial species that share dual functions within the same enzyme, the larger pterin substrate is accommodated at the same site by an induced fit mechanism [113]. The distinction between substrates also arises from a specific hydrogen bond between the pterin heterocycle, which is functionalized in the FPFG substrate, with a conserved Asp residue in the DHFS site. The DHFS enzymes have a highly conserved loop obstructing the functionalized pterin, preserving the specificity for monoglutamation [108]. The benzoyl portion of the dihydropteroate substrate is positioned at the periphery of the pterin site, which is distinctive but shared between DHFS and FPFG [108].

The reaction generates a tetrahedral intermediate by first transferring a phosphate moiety from ATP onto dihydropteroate, and that is subsequently reacted with L-glutamate. This intermediate is resolved, generating glutamylated folate and freeing an inorganic phosphate unit [108]. It was demonstrated that methotrexate, the universal DHFR inhibitor, does not inhibit DHFS [116]. A feedback mechanism has been identified, whereby buildup of the DHFR product (DHFR substrate) dihydrofolate causes inhibition of DHFS, such as in response to DHFR inhibitors [117]. Novel folate analogs that mimic the tetrahedral intermediate with a phosphinate linkage were identified [118] and the structural basis for inhibition has been validated [116].

### 2.7. Enzymes for the Biosynthesis of Folate: 7. Dihydrofolate Reductase (DHFR)

**Figure 7:**
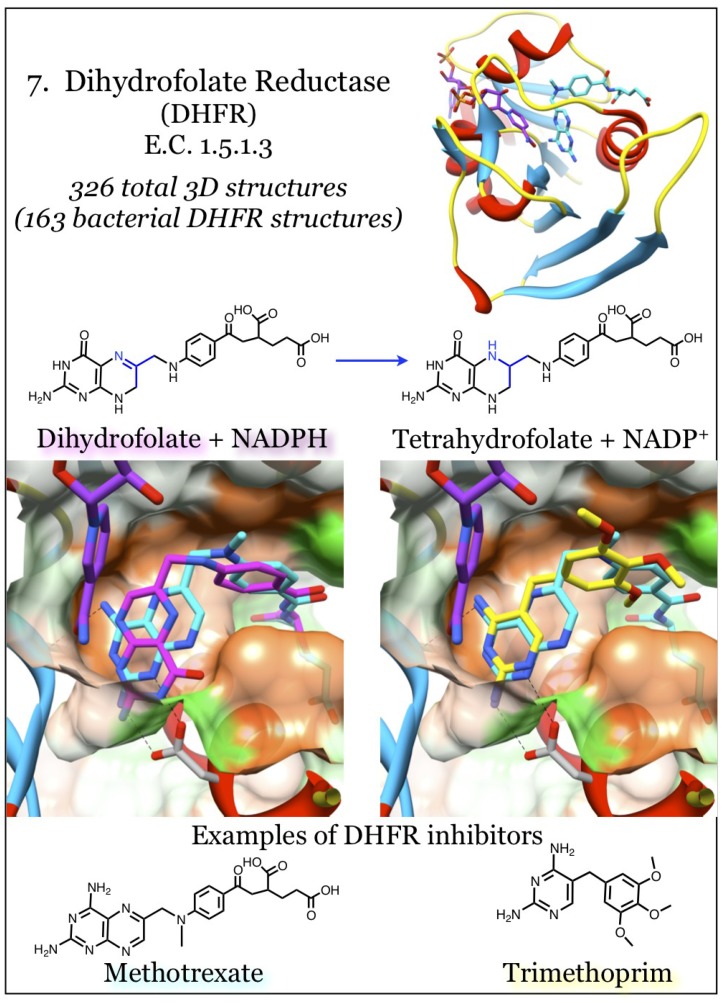
Dihydrofolate reductase (DHFR) acts on dihydrofolate to produce tetrahydrofolate using the reducing power of NADPH. Coordinates for DHFR complexed with the dihydrofolate substrate (magenta) and the NADPH co-factor (purple) were taken from PDB ID 1RF7 [120]. Coordinates for the complex with methotrexate (cyan) are from 3DAU, and those with trimethoprim (yellow) are from 3FRE [45,138]. The pterin binding pocket is fairly hydrophobic (orange surface) with limited polar areas (green surface). Conserved interactions of the aminopterin moiety, and mimics thereof, with a conserved acidic residue are indicated.

Dihydrofolate reductase (DHFR, E.C. 1.5.1.3), the most targeted member in folate metabolism, uses dihydrofolate as a substrate and reduces it to tetrahydrofolate in an NADPH-dependent reaction. Its effectiveness as a target mediating anti-proliferation effects arises from its absolute requirement for cellular metabolism. It produces tetrahydrofolate, the co-factor and donor for one-carbon transfer reactions (Figure 7). The NADP(H) co-factor wraps around the outside of the molecule, while the dihydrofolate substrate and catalytically involved nicotinamide are held adjacent in a less accessible pocket. Early work with the DHFR from *E. coli* characterized the catalytic mechanism of the enzyme [119,120]. This work highlighted a flexible loop centered on residue Met20 (*E. coli* numbering) that is responsive to co-factor binding and catalysis, and is strongly implicated in product release. Bacterial DHFR enzymes can be effectively targeted while sparing the eukaryotic DHFR due to these sequence changes [47,121]. Recent work examined the evolution of DHFR sequences and identified three crucial areas of human DHFR sequence variation and delineated their impacted on the catalytic mechanism [122]. This study revealed mutations arose to remove the dependency of the Met20 loop’s movement, thus limiting the flexibility of eukaryotic DHFR. Compensatory mutations in the eukaryotic DHFR binding site proximal to the glutamyl-tail binding site, particularly the “PEKN” loop insertion, restore the catalytic efficiency.

Inhibitors of DHFR are folate substrate mimetics, and their properties have been extensively reviewed; there are currently eight anti-folates in clinical use [34]. They can be divided into “classical” inhibitors and “non-classical” inhibitors based upon the presence of the poly-glutamate moiety. Glutamation is present in the “classical” type and is typical in anti-cancer agents as it allows active uptake of compounds into cells. Eukaryotic DHFR is a target for anti-cancer therapies such as methotrexate, which works so remarkably well that non-cancerous cells must sometimes be rescued by leucovorin (a tetrahydrofolate analog) [123,124]. Methotrexate, a substrate mimetic, inhibits all known DHFR enzymes. Its potency derives from its structural similarity to the natural substrate, dihydrofolate, with only an oxygen to nitrogen substitution at the pterin moiety (Figure 7). The pterin substituent, which is almost identical to that of folate, docks deep within the binding site and satisfies the same hydrogen bonding relationships as found with the natural substrate [28,45,50,119,125]. Methotrexate is known to interact with enzymes downstream of DHFR, but it is ineffective with the upstream enzymes needed for the biosynthesis of folate.

Antimicrobials that inhibit the DHFR enzyme belong to the “non-classical” category and rely on cell entry *via* diffusion [111]. However, they are readily susceptible to the action of efflux pumps in many Gram-negative bacteria [126,127,128,129]. Anti-folates that target fungal pathogens have been heavily pursued, particularly with regard to Acquired Immunodeficiency Syndrome (AIDS)-related complications [47,130,131,132,133,134]. Trimethoprim (TMP) is a very effective antimicrobial and its structure contains a 2,4-diaminopyrimidine moiety rather than a pterin heterocycle. The diaminopyrimidine ring is able to satisfy the hydrogen bonding criteria implicit in the folate substrate site. In general, the most promising inhibitors, as assessed by potency, maintain this pattern of hydrogen bonding, and these interactions are needed for effective inhibition [28,50]. TMP was introduced to the clinic in 1962 and resistance identified by 1968 [135]. Many more inhibitors have been extended from the 2,4-diaminopyrimidine base, including some that have entered clinical trials [136,137].

The DHFR enzymes from a variety of bacteria have been analyzed and as of 27 October 2013, more than 160 X-ray crystal structures have been deposited with the Protein Databank (see Supplemental Table S1). These have revealed a highly conserved deeper pocket with optimum hydrogen bond donors and acceptors to accommodate a pterin heterocycle or dihydropyrimidine ring, although the orientation of the di-amino moieties can be flipped within the pocket [50,139]. Mutations around this deep pocket have been demonstrated to infer resistance to trimethoprim, such as the Phe to Tyr mutation at position 98 of the *S. aureus* DHFR [140,141,142,143,144,145,146,147]. Isolates of many bacteria encode for anti-folate resistant DHFR enzymes, and these mutations cluster around and just proximal to the pterin-docking site. Further exploration of the remaining large volume in the substrate site of bacterial DHFR enzymes has revealed species-specific variations that should be useful to develop narrow-spectrum targeting strategies [131,146,148,149,150,151]. In addition, those studies highlighted the generally more hydrophobic character of the DHFR site relative to the other (upstream) members of the biosynthetic folate pathway. 

## 3. Conclusions

**Figure 8:**
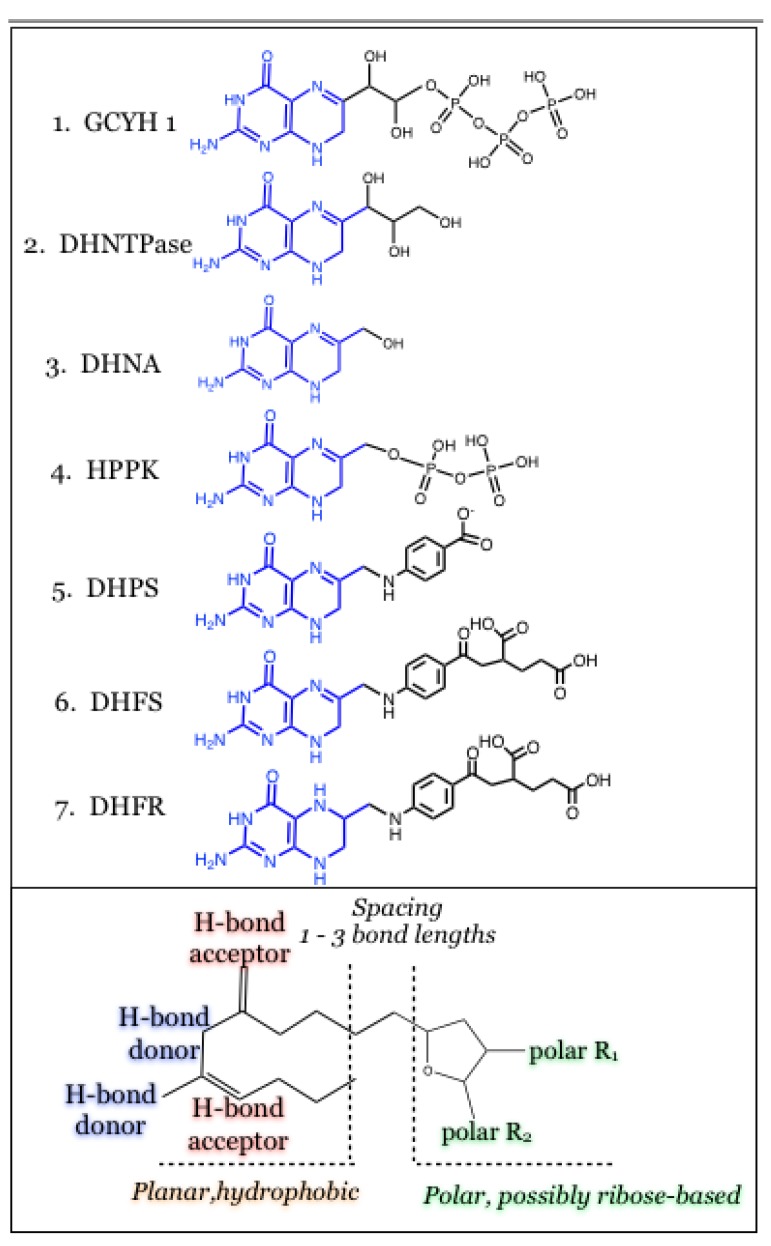
Overlap of enzyme product sub-structures provides a template pharmacophore for multi-pronged compounds. The structure of the pterin-like heterocycle is conserved in the ligands for each of the seven enzymes, and each enzyme makes similar hydrogen bonding interactions. A potential inhibitor that targets more than one of these seven enzymes should include a relatively planar and hydrophobic framework with hydrogen-bonding potential at the periphery. The ligand sub-structures from the first four enzymes maintain a relatively polar character, while the latter three ligand sub-structures are hydrophobic at the core and polar at the distal end. This can possibly be accommodated in an inhibitory compound using carbohydrate units, and its spacing from the first component should be varied by 1–3 bond lengths of neutral character.

The similarity of the natural ligands can directly inform on the structure for a similar, possibly minimalistic, inhibitor structure that can target multiple points in the pathway with the same single compound. The idea of successfully inhibiting a series of highly related enzymes in a species-specific manner seems difficult. Such a compound must maintain the specific hydrogen-bonding pattern observed with the pterin-like moiety of the folate substrates (Figure 8), and in addition should be presented in the context of a relatively planar and hydrophobic unit. Among the seven enzymes utilized for biosynthesis of folate, the first four (GCYH I, DHNTPase, DHNA, and HPPK) contain binding sites that are more polar or proximal to the protein’s surface. The latter three enzymes (DHPS, DHFS, DHFR) have deeper and more hydrophobic pockets. While these properties may distinguish the inhibitory profiles into two subsets, it may be possible to accommodate each of these using carbohydrates such as ribose sugars (Figure 8). These maintain a high polarity but are constrained within a volume or shape that would complement even the deeper binding sites. The spacing between such a moiety relatively to the hydrogen-bonded end is a variable that can only be properly addressed through experimentation. The high similarity of pterin-binding pockets within the biosynthetic folate pathway, particularly upstream of the DHFR enzyme, shows great promise for establishing proof-of-principle of this concept.

Interestingly, although anti-folates are synthetic, there is a large pool of bacteria with natural resistance to these therapies [147,152,153]. It has been proposed that antibiotic resistance arises due to selective pressure; however, recovery of resistant populations from “untouched” ecological sites either refutes this or indicates unknown pressures from folate-like compounds. If such folate-like compounds exist they would provide a new template for design of therapeutics.

## Supplementary Files

Supplementary File 1
